# Supplementary figures and images for: Towards Low-Cost Hyperspectral Single-Pixel Imaging for Plant Phenotyping (part 2 of 2)
Source: Sensors (Basel). 2020 Feb 19;20(4):1132. doi: 10.3390/s20041132 (PMC7070961; doi:10.3390/s20041132)

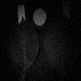

Supplement: Supplementary file 1 [file sensors-20-01132-s001.zip › Supplementary Materials/S1/S1/Hypercube_182.png]

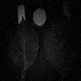

Supplement: Supplementary file 1 [file sensors-20-01132-s001.zip › Supplementary Materials/S1/S1/Hypercube_183.png]

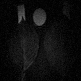

Supplement: Supplementary file 1 [file sensors-20-01132-s001.zip › Supplementary Materials/S1/S1/Hypercube_184.png]

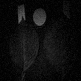

Supplement: Supplementary file 1 [file sensors-20-01132-s001.zip › Supplementary Materials/S1/S1/Hypercube_185.png]

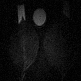

Supplement: Supplementary file 1 [file sensors-20-01132-s001.zip › Supplementary Materials/S1/S1/Hypercube_186.png]

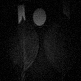

Supplement: Supplementary file 1 [file sensors-20-01132-s001.zip › Supplementary Materials/S1/S1/Hypercube_187.png]

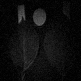

Supplement: Supplementary file 1 [file sensors-20-01132-s001.zip › Supplementary Materials/S1/S1/Hypercube_188.png]

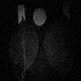

Supplement: Supplementary file 1 [file sensors-20-01132-s001.zip › Supplementary Materials/S1/S1/Hypercube_189.png]

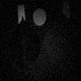

Supplement: Supplementary file 1 [file sensors-20-01132-s001.zip › Supplementary Materials/S1/S1/Hypercube_19.png]

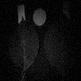

Supplement: Supplementary file 1 [file sensors-20-01132-s001.zip › Supplementary Materials/S1/S1/Hypercube_190.png]

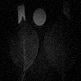

Supplement: Supplementary file 1 [file sensors-20-01132-s001.zip › Supplementary Materials/S1/S1/Hypercube_191.png]

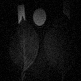

Supplement: Supplementary file 1 [file sensors-20-01132-s001.zip › Supplementary Materials/S1/S1/Hypercube_192.png]

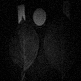

Supplement: Supplementary file 1 [file sensors-20-01132-s001.zip › Supplementary Materials/S1/S1/Hypercube_193.png]

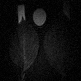

Supplement: Supplementary file 1 [file sensors-20-01132-s001.zip › Supplementary Materials/S1/S1/Hypercube_194.png]

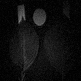

Supplement: Supplementary file 1 [file sensors-20-01132-s001.zip › Supplementary Materials/S1/S1/Hypercube_195.png]

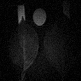

Supplement: Supplementary file 1 [file sensors-20-01132-s001.zip › Supplementary Materials/S1/S1/Hypercube_196.png]

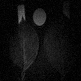

Supplement: Supplementary file 1 [file sensors-20-01132-s001.zip › Supplementary Materials/S1/S1/Hypercube_197.png]

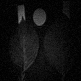

Supplement: Supplementary file 1 [file sensors-20-01132-s001.zip › Supplementary Materials/S1/S1/Hypercube_198.png]

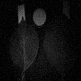

Supplement: Supplementary file 1 [file sensors-20-01132-s001.zip › Supplementary Materials/S1/S1/Hypercube_199.png]

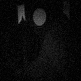

Supplement: Supplementary file 1 [file sensors-20-01132-s001.zip › Supplementary Materials/S1/S1/Hypercube_20.png]

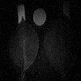

Supplement: Supplementary file 1 [file sensors-20-01132-s001.zip › Supplementary Materials/S1/S1/Hypercube_200.png]

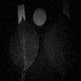

Supplement: Supplementary file 1 [file sensors-20-01132-s001.zip › Supplementary Materials/S1/S1/Hypercube_201.png]

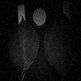

Supplement: Supplementary file 1 [file sensors-20-01132-s001.zip › Supplementary Materials/S1/S1/Hypercube_202.png]

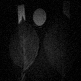

Supplement: Supplementary file 1 [file sensors-20-01132-s001.zip › Supplementary Materials/S1/S1/Hypercube_203.png]

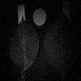

Supplement: Supplementary file 1 [file sensors-20-01132-s001.zip › Supplementary Materials/S1/S1/Hypercube_204.png]

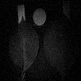

Supplement: Supplementary file 1 [file sensors-20-01132-s001.zip › Supplementary Materials/S1/S1/Hypercube_205.png]

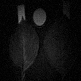

Supplement: Supplementary file 1 [file sensors-20-01132-s001.zip › Supplementary Materials/S1/S1/Hypercube_206.png]

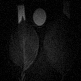

Supplement: Supplementary file 1 [file sensors-20-01132-s001.zip › Supplementary Materials/S1/S1/Hypercube_207.png]

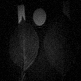

Supplement: Supplementary file 1 [file sensors-20-01132-s001.zip › Supplementary Materials/S1/S1/Hypercube_208.png]

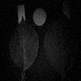

Supplement: Supplementary file 1 [file sensors-20-01132-s001.zip › Supplementary Materials/S1/S1/Hypercube_209.png]

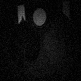

Supplement: Supplementary file 1 [file sensors-20-01132-s001.zip › Supplementary Materials/S1/S1/Hypercube_21.png]

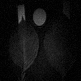

Supplement: Supplementary file 1 [file sensors-20-01132-s001.zip › Supplementary Materials/S1/S1/Hypercube_210.png]

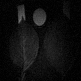

Supplement: Supplementary file 1 [file sensors-20-01132-s001.zip › Supplementary Materials/S1/S1/Hypercube_211.png]

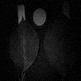

Supplement: Supplementary file 1 [file sensors-20-01132-s001.zip › Supplementary Materials/S1/S1/Hypercube_212.png]

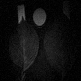

Supplement: Supplementary file 1 [file sensors-20-01132-s001.zip › Supplementary Materials/S1/S1/Hypercube_213.png]

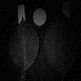

Supplement: Supplementary file 1 [file sensors-20-01132-s001.zip › Supplementary Materials/S1/S1/Hypercube_214.png]

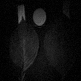

Supplement: Supplementary file 1 [file sensors-20-01132-s001.zip › Supplementary Materials/S1/S1/Hypercube_215.png]

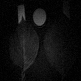

Supplement: Supplementary file 1 [file sensors-20-01132-s001.zip › Supplementary Materials/S1/S1/Hypercube_216.png]

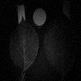

Supplement: Supplementary file 1 [file sensors-20-01132-s001.zip › Supplementary Materials/S1/S1/Hypercube_217.png]

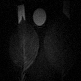

Supplement: Supplementary file 1 [file sensors-20-01132-s001.zip › Supplementary Materials/S1/S1/Hypercube_218.png]

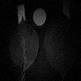

Supplement: Supplementary file 1 [file sensors-20-01132-s001.zip › Supplementary Materials/S1/S1/Hypercube_219.png]

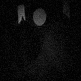

Supplement: Supplementary file 1 [file sensors-20-01132-s001.zip › Supplementary Materials/S1/S1/Hypercube_22.png]

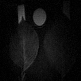

Supplement: Supplementary file 1 [file sensors-20-01132-s001.zip › Supplementary Materials/S1/S1/Hypercube_220.png]

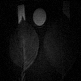

Supplement: Supplementary file 1 [file sensors-20-01132-s001.zip › Supplementary Materials/S1/S1/Hypercube_221.png]

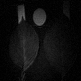

Supplement: Supplementary file 1 [file sensors-20-01132-s001.zip › Supplementary Materials/S1/S1/Hypercube_222.png]

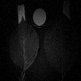

Supplement: Supplementary file 1 [file sensors-20-01132-s001.zip › Supplementary Materials/S1/S1/Hypercube_223.png]

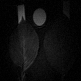

Supplement: Supplementary file 1 [file sensors-20-01132-s001.zip › Supplementary Materials/S1/S1/Hypercube_224.png]

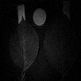

Supplement: Supplementary file 1 [file sensors-20-01132-s001.zip › Supplementary Materials/S1/S1/Hypercube_225.png]

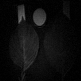

Supplement: Supplementary file 1 [file sensors-20-01132-s001.zip › Supplementary Materials/S1/S1/Hypercube_226.png]

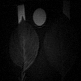

Supplement: Supplementary file 1 [file sensors-20-01132-s001.zip › Supplementary Materials/S1/S1/Hypercube_227.png]

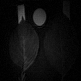

Supplement: Supplementary file 1 [file sensors-20-01132-s001.zip › Supplementary Materials/S1/S1/Hypercube_228.png]

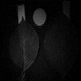

Supplement: Supplementary file 1 [file sensors-20-01132-s001.zip › Supplementary Materials/S1/S1/Hypercube_229.png]

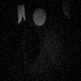

Supplement: Supplementary file 1 [file sensors-20-01132-s001.zip › Supplementary Materials/S1/S1/Hypercube_23.png]

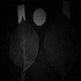

Supplement: Supplementary file 1 [file sensors-20-01132-s001.zip › Supplementary Materials/S1/S1/Hypercube_230.png]

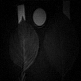

Supplement: Supplementary file 1 [file sensors-20-01132-s001.zip › Supplementary Materials/S1/S1/Hypercube_231.png]

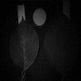

Supplement: Supplementary file 1 [file sensors-20-01132-s001.zip › Supplementary Materials/S1/S1/Hypercube_232.png]

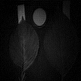

Supplement: Supplementary file 1 [file sensors-20-01132-s001.zip › Supplementary Materials/S1/S1/Hypercube_233.png]

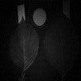

Supplement: Supplementary file 1 [file sensors-20-01132-s001.zip › Supplementary Materials/S1/S1/Hypercube_234.png]

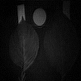

Supplement: Supplementary file 1 [file sensors-20-01132-s001.zip › Supplementary Materials/S1/S1/Hypercube_235.png]

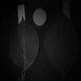

Supplement: Supplementary file 1 [file sensors-20-01132-s001.zip › Supplementary Materials/S1/S1/Hypercube_236.png]

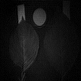

Supplement: Supplementary file 1 [file sensors-20-01132-s001.zip › Supplementary Materials/S1/S1/Hypercube_237.png]

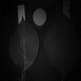

Supplement: Supplementary file 1 [file sensors-20-01132-s001.zip › Supplementary Materials/S1/S1/Hypercube_238.png]

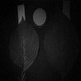

Supplement: Supplementary file 1 [file sensors-20-01132-s001.zip › Supplementary Materials/S1/S1/Hypercube_239.png]

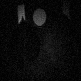

Supplement: Supplementary file 1 [file sensors-20-01132-s001.zip › Supplementary Materials/S1/S1/Hypercube_24.png]

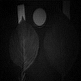

Supplement: Supplementary file 1 [file sensors-20-01132-s001.zip › Supplementary Materials/S1/S1/Hypercube_240.png]

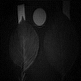

Supplement: Supplementary file 1 [file sensors-20-01132-s001.zip › Supplementary Materials/S1/S1/Hypercube_241.png]

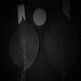

Supplement: Supplementary file 1 [file sensors-20-01132-s001.zip › Supplementary Materials/S1/S1/Hypercube_242.png]

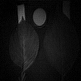

Supplement: Supplementary file 1 [file sensors-20-01132-s001.zip › Supplementary Materials/S1/S1/Hypercube_243.png]

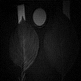

Supplement: Supplementary file 1 [file sensors-20-01132-s001.zip › Supplementary Materials/S1/S1/Hypercube_244.png]

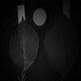

Supplement: Supplementary file 1 [file sensors-20-01132-s001.zip › Supplementary Materials/S1/S1/Hypercube_245.png]

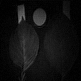

Supplement: Supplementary file 1 [file sensors-20-01132-s001.zip › Supplementary Materials/S1/S1/Hypercube_246.png]

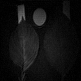

Supplement: Supplementary file 1 [file sensors-20-01132-s001.zip › Supplementary Materials/S1/S1/Hypercube_247.png]

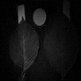

Supplement: Supplementary file 1 [file sensors-20-01132-s001.zip › Supplementary Materials/S1/S1/Hypercube_248.png]

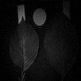

Supplement: Supplementary file 1 [file sensors-20-01132-s001.zip › Supplementary Materials/S1/S1/Hypercube_249.png]

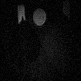

Supplement: Supplementary file 1 [file sensors-20-01132-s001.zip › Supplementary Materials/S1/S1/Hypercube_25.png]

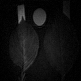

Supplement: Supplementary file 1 [file sensors-20-01132-s001.zip › Supplementary Materials/S1/S1/Hypercube_250.png]

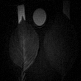

Supplement: Supplementary file 1 [file sensors-20-01132-s001.zip › Supplementary Materials/S1/S1/Hypercube_251.png]

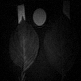

Supplement: Supplementary file 1 [file sensors-20-01132-s001.zip › Supplementary Materials/S1/S1/Hypercube_252.png]

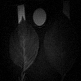

Supplement: Supplementary file 1 [file sensors-20-01132-s001.zip › Supplementary Materials/S1/S1/Hypercube_253.png]

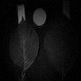

Supplement: Supplementary file 1 [file sensors-20-01132-s001.zip › Supplementary Materials/S1/S1/Hypercube_254.png]

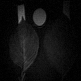

Supplement: Supplementary file 1 [file sensors-20-01132-s001.zip › Supplementary Materials/S1/S1/Hypercube_255.png]

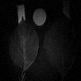

Supplement: Supplementary file 1 [file sensors-20-01132-s001.zip › Supplementary Materials/S1/S1/Hypercube_256.png]

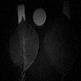

Supplement: Supplementary file 1 [file sensors-20-01132-s001.zip › Supplementary Materials/S1/S1/Hypercube_257.png]

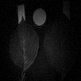

Supplement: Supplementary file 1 [file sensors-20-01132-s001.zip › Supplementary Materials/S1/S1/Hypercube_258.png]

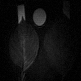

Supplement: Supplementary file 1 [file sensors-20-01132-s001.zip › Supplementary Materials/S1/S1/Hypercube_259.png]

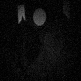

Supplement: Supplementary file 1 [file sensors-20-01132-s001.zip › Supplementary Materials/S1/S1/Hypercube_26.png]

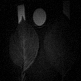

Supplement: Supplementary file 1 [file sensors-20-01132-s001.zip › Supplementary Materials/S1/S1/Hypercube_260.png]

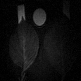

Supplement: Supplementary file 1 [file sensors-20-01132-s001.zip › Supplementary Materials/S1/S1/Hypercube_261.png]

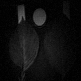

Supplement: Supplementary file 1 [file sensors-20-01132-s001.zip › Supplementary Materials/S1/S1/Hypercube_262.png]

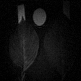

Supplement: Supplementary file 1 [file sensors-20-01132-s001.zip › Supplementary Materials/S1/S1/Hypercube_263.png]

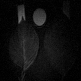

Supplement: Supplementary file 1 [file sensors-20-01132-s001.zip › Supplementary Materials/S1/S1/Hypercube_264.png]

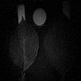

Supplement: Supplementary file 1 [file sensors-20-01132-s001.zip › Supplementary Materials/S1/S1/Hypercube_265.png]

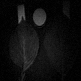

Supplement: Supplementary file 1 [file sensors-20-01132-s001.zip › Supplementary Materials/S1/S1/Hypercube_266.png]

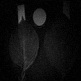

Supplement: Supplementary file 1 [file sensors-20-01132-s001.zip › Supplementary Materials/S1/S1/Hypercube_267.png]

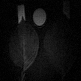

Supplement: Supplementary file 1 [file sensors-20-01132-s001.zip › Supplementary Materials/S1/S1/Hypercube_268.png]

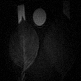

Supplement: Supplementary file 1 [file sensors-20-01132-s001.zip › Supplementary Materials/S1/S1/Hypercube_269.png]

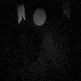

Supplement: Supplementary file 1 [file sensors-20-01132-s001.zip › Supplementary Materials/S1/S1/Hypercube_27.png]

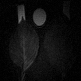

Supplement: Supplementary file 1 [file sensors-20-01132-s001.zip › Supplementary Materials/S1/S1/Hypercube_270.png]

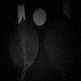

Supplement: Supplementary file 1 [file sensors-20-01132-s001.zip › Supplementary Materials/S1/S1/Hypercube_271.png]

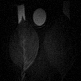

Supplement: Supplementary file 1 [file sensors-20-01132-s001.zip › Supplementary Materials/S1/S1/Hypercube_272.png]
