# Supplementary figures and images for: Towards Low-Cost Hyperspectral Single-Pixel Imaging for Plant Phenotyping (part 1 of 2)
Source: Sensors (Basel). 2020 Feb 19;20(4):1132. doi: 10.3390/s20041132 (PMC7070961; doi:10.3390/s20041132)

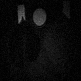

Supplement: Supplementary file 1 [file sensors-20-01132-s001.zip › Supplementary Materials/S1/S1/Hypercube_01.png]

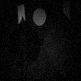

Supplement: Supplementary file 1 [file sensors-20-01132-s001.zip › Supplementary Materials/S1/S1/Hypercube_02.png]

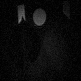

Supplement: Supplementary file 1 [file sensors-20-01132-s001.zip › Supplementary Materials/S1/S1/Hypercube_03.png]

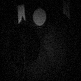

Supplement: Supplementary file 1 [file sensors-20-01132-s001.zip › Supplementary Materials/S1/S1/Hypercube_04.png]

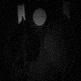

Supplement: Supplementary file 1 [file sensors-20-01132-s001.zip › Supplementary Materials/S1/S1/Hypercube_05.png]

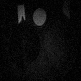

Supplement: Supplementary file 1 [file sensors-20-01132-s001.zip › Supplementary Materials/S1/S1/Hypercube_06.png]

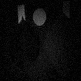

Supplement: Supplementary file 1 [file sensors-20-01132-s001.zip › Supplementary Materials/S1/S1/Hypercube_07.png]

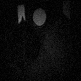

Supplement: Supplementary file 1 [file sensors-20-01132-s001.zip › Supplementary Materials/S1/S1/Hypercube_08.png]

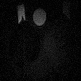

Supplement: Supplementary file 1 [file sensors-20-01132-s001.zip › Supplementary Materials/S1/S1/Hypercube_09.png]

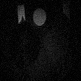

Supplement: Supplementary file 1 [file sensors-20-01132-s001.zip › Supplementary Materials/S1/S1/Hypercube_10.png]

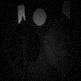

Supplement: Supplementary file 1 [file sensors-20-01132-s001.zip › Supplementary Materials/S1/S1/Hypercube_100.png]

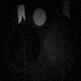

Supplement: Supplementary file 1 [file sensors-20-01132-s001.zip › Supplementary Materials/S1/S1/Hypercube_101.png]

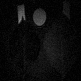

Supplement: Supplementary file 1 [file sensors-20-01132-s001.zip › Supplementary Materials/S1/S1/Hypercube_102.png]

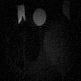

Supplement: Supplementary file 1 [file sensors-20-01132-s001.zip › Supplementary Materials/S1/S1/Hypercube_103.png]

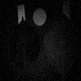

Supplement: Supplementary file 1 [file sensors-20-01132-s001.zip › Supplementary Materials/S1/S1/Hypercube_104.png]

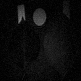

Supplement: Supplementary file 1 [file sensors-20-01132-s001.zip › Supplementary Materials/S1/S1/Hypercube_105.png]

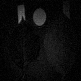

Supplement: Supplementary file 1 [file sensors-20-01132-s001.zip › Supplementary Materials/S1/S1/Hypercube_106.png]

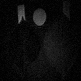

Supplement: Supplementary file 1 [file sensors-20-01132-s001.zip › Supplementary Materials/S1/S1/Hypercube_107.png]

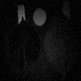

Supplement: Supplementary file 1 [file sensors-20-01132-s001.zip › Supplementary Materials/S1/S1/Hypercube_108.png]

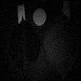

Supplement: Supplementary file 1 [file sensors-20-01132-s001.zip › Supplementary Materials/S1/S1/Hypercube_109.png]

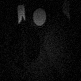

Supplement: Supplementary file 1 [file sensors-20-01132-s001.zip › Supplementary Materials/S1/S1/Hypercube_11.png]

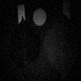

Supplement: Supplementary file 1 [file sensors-20-01132-s001.zip › Supplementary Materials/S1/S1/Hypercube_110.png]

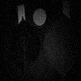

Supplement: Supplementary file 1 [file sensors-20-01132-s001.zip › Supplementary Materials/S1/S1/Hypercube_111.png]

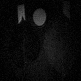

Supplement: Supplementary file 1 [file sensors-20-01132-s001.zip › Supplementary Materials/S1/S1/Hypercube_112.png]

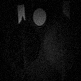

Supplement: Supplementary file 1 [file sensors-20-01132-s001.zip › Supplementary Materials/S1/S1/Hypercube_113.png]

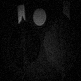

Supplement: Supplementary file 1 [file sensors-20-01132-s001.zip › Supplementary Materials/S1/S1/Hypercube_114.png]

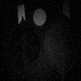

Supplement: Supplementary file 1 [file sensors-20-01132-s001.zip › Supplementary Materials/S1/S1/Hypercube_115.png]

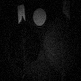

Supplement: Supplementary file 1 [file sensors-20-01132-s001.zip › Supplementary Materials/S1/S1/Hypercube_116.png]

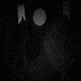

Supplement: Supplementary file 1 [file sensors-20-01132-s001.zip › Supplementary Materials/S1/S1/Hypercube_117.png]

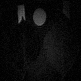

Supplement: Supplementary file 1 [file sensors-20-01132-s001.zip › Supplementary Materials/S1/S1/Hypercube_118.png]

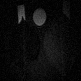

Supplement: Supplementary file 1 [file sensors-20-01132-s001.zip › Supplementary Materials/S1/S1/Hypercube_119.png]

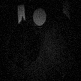

Supplement: Supplementary file 1 [file sensors-20-01132-s001.zip › Supplementary Materials/S1/S1/Hypercube_12.png]

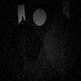

Supplement: Supplementary file 1 [file sensors-20-01132-s001.zip › Supplementary Materials/S1/S1/Hypercube_120.png]

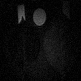

Supplement: Supplementary file 1 [file sensors-20-01132-s001.zip › Supplementary Materials/S1/S1/Hypercube_121.png]

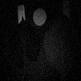

Supplement: Supplementary file 1 [file sensors-20-01132-s001.zip › Supplementary Materials/S1/S1/Hypercube_122.png]

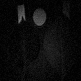

Supplement: Supplementary file 1 [file sensors-20-01132-s001.zip › Supplementary Materials/S1/S1/Hypercube_123.png]

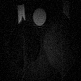

Supplement: Supplementary file 1 [file sensors-20-01132-s001.zip › Supplementary Materials/S1/S1/Hypercube_124.png]

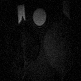

Supplement: Supplementary file 1 [file sensors-20-01132-s001.zip › Supplementary Materials/S1/S1/Hypercube_125.png]

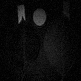

Supplement: Supplementary file 1 [file sensors-20-01132-s001.zip › Supplementary Materials/S1/S1/Hypercube_126.png]

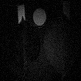

Supplement: Supplementary file 1 [file sensors-20-01132-s001.zip › Supplementary Materials/S1/S1/Hypercube_127.png]

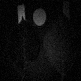

Supplement: Supplementary file 1 [file sensors-20-01132-s001.zip › Supplementary Materials/S1/S1/Hypercube_128.png]

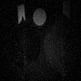

Supplement: Supplementary file 1 [file sensors-20-01132-s001.zip › Supplementary Materials/S1/S1/Hypercube_129.png]

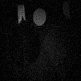

Supplement: Supplementary file 1 [file sensors-20-01132-s001.zip › Supplementary Materials/S1/S1/Hypercube_13.png]

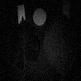

Supplement: Supplementary file 1 [file sensors-20-01132-s001.zip › Supplementary Materials/S1/S1/Hypercube_130.png]

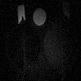

Supplement: Supplementary file 1 [file sensors-20-01132-s001.zip › Supplementary Materials/S1/S1/Hypercube_131.png]

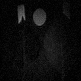

Supplement: Supplementary file 1 [file sensors-20-01132-s001.zip › Supplementary Materials/S1/S1/Hypercube_132.png]

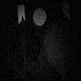

Supplement: Supplementary file 1 [file sensors-20-01132-s001.zip › Supplementary Materials/S1/S1/Hypercube_133.png]

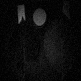

Supplement: Supplementary file 1 [file sensors-20-01132-s001.zip › Supplementary Materials/S1/S1/Hypercube_134.png]

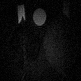

Supplement: Supplementary file 1 [file sensors-20-01132-s001.zip › Supplementary Materials/S1/S1/Hypercube_135.png]

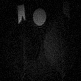

Supplement: Supplementary file 1 [file sensors-20-01132-s001.zip › Supplementary Materials/S1/S1/Hypercube_136.png]

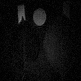

Supplement: Supplementary file 1 [file sensors-20-01132-s001.zip › Supplementary Materials/S1/S1/Hypercube_137.png]

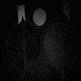

Supplement: Supplementary file 1 [file sensors-20-01132-s001.zip › Supplementary Materials/S1/S1/Hypercube_138.png]

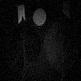

Supplement: Supplementary file 1 [file sensors-20-01132-s001.zip › Supplementary Materials/S1/S1/Hypercube_139.png]

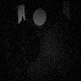

Supplement: Supplementary file 1 [file sensors-20-01132-s001.zip › Supplementary Materials/S1/S1/Hypercube_14.png]

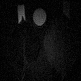

Supplement: Supplementary file 1 [file sensors-20-01132-s001.zip › Supplementary Materials/S1/S1/Hypercube_140.png]

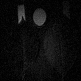

Supplement: Supplementary file 1 [file sensors-20-01132-s001.zip › Supplementary Materials/S1/S1/Hypercube_141.png]

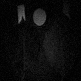

Supplement: Supplementary file 1 [file sensors-20-01132-s001.zip › Supplementary Materials/S1/S1/Hypercube_142.png]

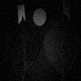

Supplement: Supplementary file 1 [file sensors-20-01132-s001.zip › Supplementary Materials/S1/S1/Hypercube_143.png]

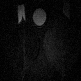

Supplement: Supplementary file 1 [file sensors-20-01132-s001.zip › Supplementary Materials/S1/S1/Hypercube_144.png]

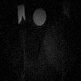

Supplement: Supplementary file 1 [file sensors-20-01132-s001.zip › Supplementary Materials/S1/S1/Hypercube_145.png]

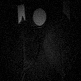

Supplement: Supplementary file 1 [file sensors-20-01132-s001.zip › Supplementary Materials/S1/S1/Hypercube_146.png]

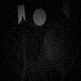

Supplement: Supplementary file 1 [file sensors-20-01132-s001.zip › Supplementary Materials/S1/S1/Hypercube_147.png]

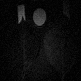

Supplement: Supplementary file 1 [file sensors-20-01132-s001.zip › Supplementary Materials/S1/S1/Hypercube_148.png]

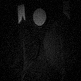

Supplement: Supplementary file 1 [file sensors-20-01132-s001.zip › Supplementary Materials/S1/S1/Hypercube_149.png]

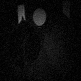

Supplement: Supplementary file 1 [file sensors-20-01132-s001.zip › Supplementary Materials/S1/S1/Hypercube_15.png]

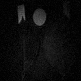

Supplement: Supplementary file 1 [file sensors-20-01132-s001.zip › Supplementary Materials/S1/S1/Hypercube_150.png]

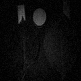

Supplement: Supplementary file 1 [file sensors-20-01132-s001.zip › Supplementary Materials/S1/S1/Hypercube_151.png]

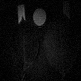

Supplement: Supplementary file 1 [file sensors-20-01132-s001.zip › Supplementary Materials/S1/S1/Hypercube_152.png]

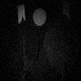

Supplement: Supplementary file 1 [file sensors-20-01132-s001.zip › Supplementary Materials/S1/S1/Hypercube_153.png]

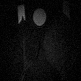

Supplement: Supplementary file 1 [file sensors-20-01132-s001.zip › Supplementary Materials/S1/S1/Hypercube_154.png]

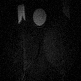

Supplement: Supplementary file 1 [file sensors-20-01132-s001.zip › Supplementary Materials/S1/S1/Hypercube_155.png]

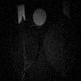

Supplement: Supplementary file 1 [file sensors-20-01132-s001.zip › Supplementary Materials/S1/S1/Hypercube_156.png]

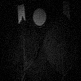

Supplement: Supplementary file 1 [file sensors-20-01132-s001.zip › Supplementary Materials/S1/S1/Hypercube_157.png]

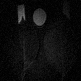

Supplement: Supplementary file 1 [file sensors-20-01132-s001.zip › Supplementary Materials/S1/S1/Hypercube_158.png]

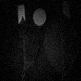

Supplement: Supplementary file 1 [file sensors-20-01132-s001.zip › Supplementary Materials/S1/S1/Hypercube_159.png]

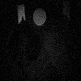

Supplement: Supplementary file 1 [file sensors-20-01132-s001.zip › Supplementary Materials/S1/S1/Hypercube_16.png]

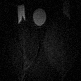

Supplement: Supplementary file 1 [file sensors-20-01132-s001.zip › Supplementary Materials/S1/S1/Hypercube_160.png]

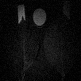

Supplement: Supplementary file 1 [file sensors-20-01132-s001.zip › Supplementary Materials/S1/S1/Hypercube_161.png]

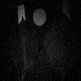

Supplement: Supplementary file 1 [file sensors-20-01132-s001.zip › Supplementary Materials/S1/S1/Hypercube_162.png]

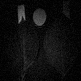

Supplement: Supplementary file 1 [file sensors-20-01132-s001.zip › Supplementary Materials/S1/S1/Hypercube_163.png]

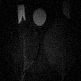

Supplement: Supplementary file 1 [file sensors-20-01132-s001.zip › Supplementary Materials/S1/S1/Hypercube_164.png]

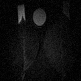

Supplement: Supplementary file 1 [file sensors-20-01132-s001.zip › Supplementary Materials/S1/S1/Hypercube_165.png]

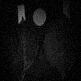

Supplement: Supplementary file 1 [file sensors-20-01132-s001.zip › Supplementary Materials/S1/S1/Hypercube_166.png]

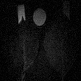

Supplement: Supplementary file 1 [file sensors-20-01132-s001.zip › Supplementary Materials/S1/S1/Hypercube_167.png]

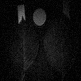

Supplement: Supplementary file 1 [file sensors-20-01132-s001.zip › Supplementary Materials/S1/S1/Hypercube_168.png]

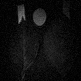

Supplement: Supplementary file 1 [file sensors-20-01132-s001.zip › Supplementary Materials/S1/S1/Hypercube_169.png]

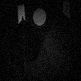

Supplement: Supplementary file 1 [file sensors-20-01132-s001.zip › Supplementary Materials/S1/S1/Hypercube_17.png]

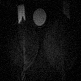

Supplement: Supplementary file 1 [file sensors-20-01132-s001.zip › Supplementary Materials/S1/S1/Hypercube_170.png]

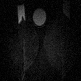

Supplement: Supplementary file 1 [file sensors-20-01132-s001.zip › Supplementary Materials/S1/S1/Hypercube_171.png]

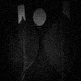

Supplement: Supplementary file 1 [file sensors-20-01132-s001.zip › Supplementary Materials/S1/S1/Hypercube_172.png]

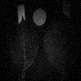

Supplement: Supplementary file 1 [file sensors-20-01132-s001.zip › Supplementary Materials/S1/S1/Hypercube_173.png]

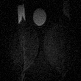

Supplement: Supplementary file 1 [file sensors-20-01132-s001.zip › Supplementary Materials/S1/S1/Hypercube_174.png]

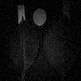

Supplement: Supplementary file 1 [file sensors-20-01132-s001.zip › Supplementary Materials/S1/S1/Hypercube_175.png]

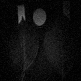

Supplement: Supplementary file 1 [file sensors-20-01132-s001.zip › Supplementary Materials/S1/S1/Hypercube_176.png]

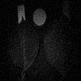

Supplement: Supplementary file 1 [file sensors-20-01132-s001.zip › Supplementary Materials/S1/S1/Hypercube_177.png]

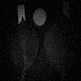

Supplement: Supplementary file 1 [file sensors-20-01132-s001.zip › Supplementary Materials/S1/S1/Hypercube_178.png]

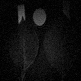

Supplement: Supplementary file 1 [file sensors-20-01132-s001.zip › Supplementary Materials/S1/S1/Hypercube_179.png]

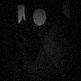

Supplement: Supplementary file 1 [file sensors-20-01132-s001.zip › Supplementary Materials/S1/S1/Hypercube_18.png]

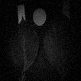

Supplement: Supplementary file 1 [file sensors-20-01132-s001.zip › Supplementary Materials/S1/S1/Hypercube_180.png]

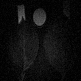

Supplement: Supplementary file 1 [file sensors-20-01132-s001.zip › Supplementary Materials/S1/S1/Hypercube_181.png]
